# Dietary Supplement Use among Primary Health Care Attendants in Abha City, Southwestern Saudi Arabia

**DOI:** 10.3390/nu13092968

**Published:** 2021-08-26

**Authors:** Safar Abadi Alsaleem, Maryam Mohammed Asiri, Mohammed Abadi Alsaleem, Abdulrahman Nasser AlShahrani, Khalid Amer Alamer, Ahmed A. Mahfouz

**Affiliations:** 1 Department of Family and Community Medicine, College of Medicine, King Khalid University, Abha 61421, Saudi Arabia; asalslim@kku.edu.sa (S.A.A.); mabade@kku.edu.sa (M.A.A.); 2 Family Medicine Department, Aseer General Directorate of Health Affairs, Abha 62523, Saudi Arabia; Drmeme1413@gmail.com; 3 College of Pharmacy, King Khalid University, Abha 62529, Saudi Arabia; 436801045@kku.edu.sa (A.N.A.); 436801137@kku.edu.sa (K.A.A.)

**Keywords:** dietary supplements, primary health care attendant, Saudi Arabia

## Abstract

Dietary supplements are commercially available manufactured products used as an addition to the normal diet and involve vitamins, minerals, herbs (botanicals), amino acids, and various other products. With the use of a cross-sectional survey, the present paper intended to analyze dietary supplement usage and its health and sociodemographic determinants among attendants of primary health care centers in Abha City, southwestern Saudi Arabia. The sample was selected randomly using the two-stage cluster sampling technique. The study included 438 participants (115 males and 323 females). Their ages ranged from 18 to 59 years, with an average of 36.2 ± 11.7 years. The study showed that 330 people used dietary supplementation, giving a prevalence of 75.3% (95% CI: 71.1–79.2%). The most commonly used supplements were multivitamins (215, 65.2%), specific vitamins (60, 18.2%), and mineral pills (38, 11.5%). Advice from health care workers was the most frequent reason for using dietary supplements (49.4%). The majority (71.2%) reported feeling a better quality of life after using dietary supplements. The most frequent disadvantages of using dietary supplements were constipation and headache (30%) and the most frequent advantage was increasing appetite (59.8%). The people who most frequently recommended the use of supplements were health care workers (190, 57.6%), followed by friends or family members (62, 18.8%), and people on social media (43, 13%). Females had a significantly higher probability of using dietary supplements than males did (Cor = 2.0, 95% CI = 1.21–3.27), and those with a chronic disease had a considerably higher likelihood of using dietary supplements (cOR = 3.48, 95% CI = 2.04–6.06). Age, educational level, and marital status were not significantly related with dietary supplement usage. In conclusion, health care workers should focus on females and persons with chronic diseases in their practice. They should provide them with evidence-based advice regarding the use of dietary supplements. Continued medical education training programs tailored to the needs of health care staff addressing this issue should be provided. New guidelines should be developed to help health professionals to provide their patients with comprehensive care at the primary health care level.

## 1. Introduction

Dietary supplements are commercially available manufactured products used as an addition to the normal diet when taken by mouth as a pill, capsule, tablet, or liquid [1]. Nutrients within the supplement are either obtained from food resources, artificially, or in combination to obtain more nutrients [2]. Vitamins, minerals, fibers, fatty acids, and amino acids are the main components of supplements [3]. Dietary supplements can also contain materials that have not been established as vital to life but are promoted as having a useful biological outcome, such as plant pigments or polyphenols [4]. In the United States and Canada, dietary supplements are regarded as a subgroup of foods and are controlled accordingly. The European Commission has also created matched guidelines to help guarantee that food supplements are safe and adequately labeled [5].

Comprising an industry estimated to have a 2015 value of USD 37 billion, more than 50,000 dietary supplement products are sold in the United States [6], and approximately half of the American adult population uses nutritional supplements. Multivitamins are the most frequently consumed product [7]. For those who do not consume a balanced diet, the United States National Institutes of Health declared that some supplements “may have value” [8].

The most frequently used supplements are multivitamins/multiminerals, vitamin D, and vitamin C [8]. The most consumed non-multivitamin/multimineral products are fish oil, omega-3/DHA, glucosamine, echinacea, flaxseed oil, chondroitin, and ginseng [9]. Dietary supplements are an essential source of nutrients that help repair any deficiencies [10].

The use of dietary supplements is more common in older individuals [11], but recently, they are becoming more used among younger individuals (<25 years of age). Athletes and gym users take different types of protein supplements to improve their muscle mass [12].

Data regarding dietary supplement usage in southwestern Saudi Arabia are scarce and even lacking. The present paper intended to analyze dietary supplement usage and its health and sociodemographic determinants among attendants of primary health care centers in Abha City, southwestern Saudi Arabia.

## 2. Materials and Methods

### 2.1. Study Design and Participants

A descriptive cross-sectional study was conducted among attendants of primary health care centers (PHCCs) in Abha City, the capital of the Aseer Region, southwestern Saudi Arabia. The city is situated 2270 m (7450 feet) above sea level. Attendants aged 18 years or more with permanent residence in Abha City (for more than one year) were included.

### 2.2. Sample Size and Sampling Technique

With an anticipated proportion of 50% and an absolute precision of 5% at the 95% confidence level, the sample size desired for the survey was calculated to be 385 persons [1]. The response level is the expected occurrence of the outcome or event of concern. Data regarding expected prevalence rates should usually be taken from the literature. When this information is not accurately obtainable, an increasing sample size is used, which is 50% [2]. A sample size of 400 persons was planned for the study to account for potential nonresponse.

The sample was selected randomly using the two-stage cluster sample technique. At the first stage, ten PHCCs in Abha City were selected randomly, covering all sectors of Abha. In the second stage, 40 participants from each selected center were randomly included using a systematic random sampling technique, including each 5th attendant.

### 2.3. Data Collection

Data were collected using a pre-structured questionnaire. The researchers developed the questionnaire after an intensive literature review and expert discussion. The questionnaire covered the following data: participants’ sociodemographic data, including age, gender, educational level, marital status, residence, job title, and monthly income; medical history and co-morbidities; and supplement use data, including if using (yes, no) and type of supplement (multivitamin and mineral pill/specific vitamin/fortified dietary substance). The reason for supplement intake (prescribed with other medications/beauty blogs/increased health awareness/gymnasium) and who recommended it (health care worker/online media/fitness trainer/friends and family/other) as well as changes perceived in quality of life (better/same as before/worse/not sure) were also included. Awareness regarding the benefits and drawbacks of using dietary supplements was included in the last section of the study questionnaire (open-ended). A pilot study including 20 participants was conducted to confirm the tool’s validity, to test for reliability, and to assess the tool’s clarity and the time to complete questionnaire. The calculated Cronbach’s alpha was 0.83 and the interitem correlation was 0.71.

### 2.4. Statistical Analysis

Categorical variables are presented as proportions (number and percentage) and continuous variables as means and standard deviations (SD). Pearson’s chi-squared test was used to assess differences in supplement usage among distinct categories of health and sociodemographic characteristics. Data were analyzed using IBM SPSS version 22 (SPSS, Inc. Chicago, IL, USA). The level of statistical significance was set at *p* < 0.05.

## 3. Results

### 3.1. Description of the Study Sample

The survey comprised 438 participants (115 males and 323 females) attending primary health care centers in Abha City, southwestern Saudi Arabia. Their ages ranged from 18 to 59 years old, with an average of 36.2 ± 11.7 years. The majority of participants (*n* = 231; 52.7%) were graduated and married (*n* = 318; 72.6%). Regarding chronic health problems, 183 individuals (41.8%) reported having a chronic disease, including diabetes (*n* = 47), hypercholesterolemia (*n* = 44), bronchial asthma (*n* = 33), and hypertension (*n* = 29).

### 3.2. Prevalence, Pattern, and Perception of Dietary Supplement Use

The study showed that 330 people used dietary supplementation, representing a prevalence of 75.3% (95% CI: 71.1–79.2%). Table 1 shows that the most commonly used supplements are multivitamins (215, 65.2%), specific vitamins (60, 18.2%), and mineral pills (38, 11.5%). Advice from health care workers was the most frequent reason for using dietary supplements (163, 49.4%), followed by improved awareness of the importance of using supplements (136, 41.2%). Similarly, the people who most frequently recommended the use of supplements were health care workers (190, 57.6%), followed by friends or family members (62, 18.8%), and people on social media (43, 13%). As for the observed changes in quality of life after using dietary supplements, the majority of the respondents (235, 71.2%) reported feeling better. Regarding the reported disadvantages of using dietary supplements, the most frequent items were constipation and headache (99, 30%), increased weight (42, 12.7%), and possible interaction with other drugs (41, 12.4%). It is worth mentioning that 105 persons (31.8%) said no disadvantages were reported. Considering the advantages of supplement use, increase in appetite was the most registered item (55, 59.8%), followed by improving immunity (48, 52.2%), and improving personal health and activity (38, 41.3%).

### 3.3. Dietary Supplement Use by Participant Background

Table 2 shows the distribution of dietary supplement use by participants’ back-ground data. Females (255, 77.3%) were using dietary supplements significantly (*p* = 0.03) more than males were (68, 63.0%). Females had a significantly higher probability of using dietary supplements than males did (cOR = 2.0, 95% CI = 1.21–3.27). Similarly, supplement use was significantly different according to whether chronic disease was present or not (*p* = 0.001). Those with chronic diseases had a significantly higher probability of using dietary supplements (cOR = 3.48, 95% CI = 2.04–6.06). On the other hand, age, educational level, and marital status were not statistically different among supplement users and non-users.

## 4. Discussion

The present study revealed that three out of four people use dietary supplements (75.3%, 95% CI: 71.1–79.2%). A similar high figure of 71.4% was reported in Kuwait [1]. A study in Lebanon showed that more than half of the participants (53%) used dietary supplements [2]. In Dubai, a study [3] assessed the utilization rate of health supplements and found a rate of 34.2% [4]. Similarly, another study in Croatia reported a figure of 30.4% [5]. Furthermore, a survey in the USA including participants from Hawaii and Los Angeles, California, showed that dietary supplement use amounted to 44% among Hawaiian males and 75% among Japanese American and White females [6]. Likewise, in Saudi Arabia, high figures were reported, amounting to 76.6% among female college students in Riyadh [7], 46.8% among pharmacy students [8], 30% among Dammam university students [9], and 26.2% among adolescent school children [10].

The current study disclosed that the most commonly used supplements were multivitamins (215, 65.2%), specific vitamins (60, 18.2%), and mineral pills (38, 11.5%). These findings are similar to those from a survey among university students in the USA, which reported that the most common supplements were multivitamin/minerals (42%) [11]. Multivitamins/minerals (56.8%) were the most frequently used among Mediterranean people [12]. Similar findings were also reported in Gulf countries, including Kuwait [1], the United Arab Emirates [3], and Saudi Arabia [7,8,9]. Advice from health care workers was the most frequently cited reason for using dietary supplements. Similar findings were reported in the United Arab Emirates [3]. Confidence in the advice of health care providers is the main reason behind this issue. The majority of participants also reported feeling a better quality of life after using dietary supplements. The most frequently reported disadvantage of using dietary supplements was constipation with headache, and the most frequent advantage was increasing appetite. Similar findings were reported in the United Arab Emirates [3], Kuwait [1], and Lebanon [2].

Our study revealed that females had a significantly higher probability of using dietary supplements than males did (cOR = 2.0, 95% CI = 1.21–3.27). The same findings were also reported in Riyadh [13], Kuwait [1], and Lebanon [2]. On the other hand, in the United Arab Emirates, dietary supplement consumption was considerably more prevalent among men than women. At the same time, men favored protein supplements (29.4%), while women used multivitamins and mineral supplements. Dietary supplement consumption was correlated to physical activity and the presence of a health issue. Males used nutritional supplements to build up their muscle bulk while females used them to improve general well-being [4].

Our findings show that those with chronic diseases have a significantly higher probability of using dietary supplements (cOR = 3.48, 95% CI = 2.04–6.06). Likewise, a survey in Canada stated that people with chronic diseases were more likely to use vitamin and mineral supplements than those free of a chronic condition [14]. Similar findings were reported in the USA [15], Malaysia [16,17], Croatia [18], and India [19].

The present study showed that half of the dietary supplement users participating in the study mentioned that the main reason for their use was based on advice from health care staff (49.4%), who were the main people recommending the service (57.6%).

A systematic review including 76 studies found that the critical factor motivating people to use dietary supplements was advice from a health care professional [20]. Health care experts’ guidance concerning the consumption of nutraceuticals was desired for most users, whether it was considered a vital factor when considering taking nutraceuticals [21], the reason for taking [22], and the reason for not taking nutraceuticals [23].

As advice from health care professionals is the leading decisive aspect in the consumption of nutraceuticals, they should attempt to use trustworthy clinical evidence-based information to help users make informed decisions about using nutraceuticals. Health care professionals should have good knowledge of dietary supplements to better care for their patients who are consuming these products. If equipped with additional in-depth information on supplements, health care professionals can better help patients to consider the possible advantages and hazards. Should adverse consequences happen, health care givers should direct patients to describe these in order to render the dietary supplement marketplace more secure [24]. Health care workers in Abha City PHCCs should focus on females and people with chronic diseases. They should provide them with evidence-based advice regarding the use of dietary supplements. New guidelines should be developed to help health professionals provide their patients with comprehensive care at the primary health care level. A limitation of this study is its cross-sectional design, which does not allow establishment of a cause–effect relationship. Furthermore, the study only addresses a localized geographical area. Additionally, the fact that a high percentage of the sample was female could have influenced the results presented.

## 5. Conclusions

In conclusion, the current study shows that nearly three out of four people use dietary supplements. The most commonly used supplements are multivitamins. Advice from health care workers was the most frequently cited reason for using dietary supplements. The majority of participants reported feeling a better quality of life after using dietary supplements. The most frequent disadvantages of using dietary supplements were constipation and headache, and the most frequent advantage was increase in appetite. Females and those with chronic diseases had a significantly higher probability of using nutritional supplements. Age, educational level, and marital status were not significantly related with dietary supplement usage. Primary health care workers in Abha City should pay more attention to their practice on females and people with chronic diseases. They should provide them with evidence-based advice regarding the use of dietary supplements. Further studies are needed to assess primary health care staff’s current knowledge and practices regarding the prescription of dietary supplements. Continued medical education training programs tailored to the needs of health care staff addressing this issue should be provided, and new guidelines should be developed to help health professionals provide their patients with comprehensive care at the primary health care level.

## Figures and Tables

**Table 1:** Pattern and perception of use of dietary supplements among users (330) attending PHCCs in Abha, Saudi Arabia.

Pattern and Perception of Supplement Use		*n*	%
Types of supplements used	Multivitamins	215	65.2%
	Specific vitamins	60	18.2%
	Mineral pills	38	11.5%
	Documented nutrients	11	3.3%
	Amino acids	6	1.8%
Reasons for using supplements	Advice from health care staff	163	49.4%
	Improved awareness	136	41.2%
	Vitamin/mineral deficiency	12	3.6%
	Others	13	3.9%
	Increase immunity	3	0.9%
	Pregnancy	3	0.9%
Who recommended using supplements	Health care staff	190	57.6%
	Specialized physician	18	5.5%
	Social and mass media	43	13.0%
	Family and friends	62	18.8%
	Others	17	5.2%
Changes in quality of life after using supplements	No change	46	13.9%
	Now worse	9	2.7%
	Now better	235	71.2%
	Not sure	40	12.1%
Disadvantages of use	None	105	31.8%
	Expensive	31	9.4%
	Increases weight	42	12.7%
	Constipation with headache	99	30.0%
	Interaction with other drugs	41	12.4%
	Causes gastritis	12	3.6%
Advantages of use	Improves immunity	48	52.2%
	Useful for hair and skin	35	38.0%
	Improves personal health and activity	38	41.3%
	Improves anemia	26	28.3%
	Increases appetite	55	59.8%

**Table 2:** Distribution of dietary supplement use by participants’ background.

Background		Users (330)		Non-Users (108)		*p*
		No.	%	No.	%	
Age in years	18–25	60	18.1%	17	15.7%	0.789
	26–35	109	33.0%	38	35.2%	
	36–45	93	28.1%	35	32.4%	
	46–50	49	14.8%	12	11.1%	
	>50 years	19	6.0%	6	5.6%	
Gender	Male	75	22.7%	40	37.0%	0.003 *
	Female	255	77.3%	68	63.0%	
Educational level	Illiterate	14	4.2%	5	4.6%	0.862
	Secondary or less	135	40.9%	47	43.5%	
	University/more	181	54.9%	56	51.9%	
Marital status	Single	77	23.3%	24	22.2%	0.896
	Married	238	72.1%	80	74.1%	
	Divorced/widow	15	4.6%	4	3.7%	
Chronic health problems	No	170	51.5%	85	78.7%	0.001 *
	Yes	160	48.5%	23	21.301	

*p*: Pearson X^2^ test; * *p* < 0.05 (significant).

## Data Availability

The data presented in this study are available on request from the corresponding author.
